# Electrophysiological network predicts clinical response to vigabatrin in epileptic spasms

**DOI:** 10.3389/fneur.2023.1209796

**Published:** 2023-06-23

**Authors:** Junhyung Kim, Min-Jee Kim, Hyun-Jin Kim, Mi-Sun Yum, Tae-Sung Ko

**Affiliations:** ^1^Department of Neurosurgery, Asan Medical Center, Seoul, Republic of Korea; ^2^Department of Pediatrics, Asan Medical Center Children’s Hospital, University of Ulsan College of Medicine, Seoul, Republic of Korea

**Keywords:** infantile spasms, West syndrome, electroencephalography, functional connectivity network, weighted phase lag index (wPLI)

## Abstract

**Purpose:**

This study aimed to discover electrophysiologic markers correlated with clinical responses to vigabatrin-based treatment in infants with epileptic spasms (ES).

**Method:**

The study involved a descriptive analysis of ES patients from a single institution, as well as electroencephalogram (EEG) analyses of 40 samples and 20 age-matched healthy infants. EEG data were acquired during the interictal sleep state prior to the standard treatment. The weighted phase-lag index (wPLI) functional connectivity was explored across frequency and spatial domains, correlating these results with clinical features.

**Results:**

Infants with ES exhibited diffuse increases in delta and theta power, differing from healthy controls. For the wPLI analysis, ES subjects exhibited higher global connectivity compared to control subjects. Subjects who responded favorably to treatment were characterized by higher beta connectivity in the parieto-occipital regions, while those with poorer outcomes exhibited lower alpha connectivity in the frontal regions. Individuals with structural neuroimaging abnormalities exhibited correspondingly low functional connectivity, implying that ES patients who maintain adequate structural and functional integrity are more likely to respond favorably to vigabatrin-based treatments.

**Conclusion:**

This study highlights the potential utility of EEG functional connectivity analysis in predicting early response to treatments in infants with ES.

## 1. Introduction

Infantile epileptic spasms syndrome, also known as epileptic spasms (ES), is one of the most hazardous neurologic conditions for infants. Its prognosis can be catastrophic, leading to severe developmental delay and intractable epilepsy. Early seizure control in ES is thought to be particularly important for their developmental outcomes (1). The current standard treatments for ES include adrenocorticotropic hormone (ACTH), prednisolone, and vigabatrin (2). While hormonal therapy has been shown to be superior to vigabatrin for ES cases without tuberous sclerosis complex (TSC), the high cost of ACTH makes practitioners hesitate to prescribe it. In source-limited clinical settings with low coverage of national health insurance, vigabatrin is still preferred as the first-line treatment (3). Regardless of the treatment regimen used, clinical response to the suppression of spasms has been believed to be important for a better prognosis (4). ES consists of a wide variety of conditions caused by numerous etiologies (5), and some subpopulations, including cases with TSC, still show favorable outcomes in response to vigabatrin monotherapy. However, recent studies have primarily concentrated on the ACTH response in ES.

Neurophysiological researchers have been interested in finding reliable predictors of treatment response based on the results of electroencephalogram (EEG) analyses. Although ES has a typical electrographic pattern known as hypsarrhythmia, the irregular and unpredictable nature of this hypsarrhythmic pattern on EEG poses challenges in risk stratification for ES when relying solely on visual EEG assessment. Several studies have investigated specific features or computational metrics that correlate with clinical outcome (6). In addition, functional connectivity networks have recently emerged as important analytics that provide valuable insights beyond the visual EEG findings (7).

In this paper, we present our perspectives on the neurophysiological basis of ES as determined using EEG analyses. We hypothesize that certain EEG-derived functional connectivity features may identify candidates likely to elicit a favorable clinical response to current standard treatments.

## 2. Materials and methods

### 2.1. Study population

**Figure 1:**
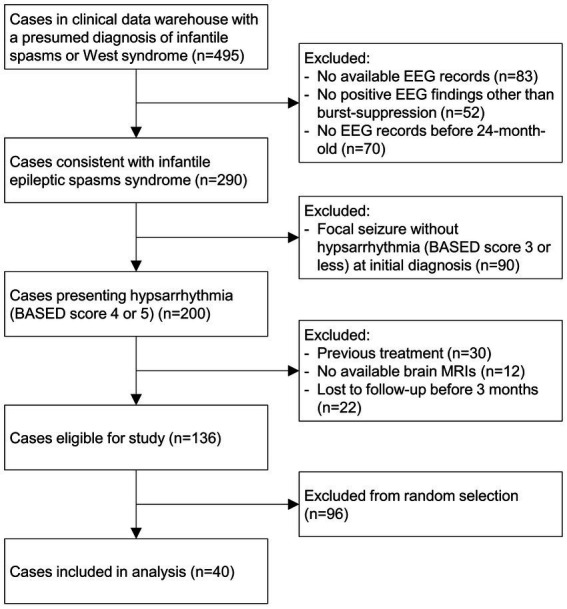
Selection of the study population. EEG, electroencephalogram, BASED, Burden of Amplitudes and Epileptiform Discharges, MRI, magnetic resonance image.

This retrospective study aimed to investigate the clinical and electrophysiological characteristics of ES cases. The study cohort was retrieved from the institutional clinical data warehouse of a tertiary-level institution (Figure 1), and all cases consistent with infantile epileptic spasms syndrome according to the 2022 ILAE (International League Against Epilepsy) classification were included. We selected cases with a formal EEG report by pediatric epileptologists indicating hypsarrhythmia with a BASED (Burden of Amplitudes and Epileptiform Discharges) score of 4 or 5. Those who were previously exposed to vigabatrin before the electrographic diagnosis, those with missing brain magnetic resonance images (MRIs) for etiologic evaluation, or those lost to follow-up before at least three months following the treatment were excluded. A group of healthy infants was additionally retrieved from the clinical data warehouse for a negative control. These cases included patients who had undergone an EEG study but had never been diagnosed with ES or other epileptic syndromes. The study was approved by the institutional review board, and informed consent was waived for all study subjects.

### 2.2. Clinical assessment

Important clinical factors that impacted ES outcomes, including etiology and initial treatment regimens, were reviewed for each individual subject. Standard treatment regimens included vigabatrin treatment, starting with a daily dose of 50 mg/kg and increasing up to 100–150 mg/kg daily, and hormonal therapy with oral prednisolone at 2 mg/kg, with or without a three-day course of methylprednisolone pulse therapy at 30 mg/kg. The clinical outcomes of interest included the suppression of spasms, intractable epilepsy, and evolution into Lennox–Gastaut syndrome. The short-term clinical response to treatment was mainly assessed at 2–4 weeks from the time of initial treatment, and the treatment was maintained for at least 3–6 months. Cases were labeled as having a favorable response (i.e., ‘responders’) if they exhibited clinical and/or electrographic suppression of spasms during the corresponding period. Other cases (i.e., ‘non-responders’) included cases that showed insufficient control of spasms and required additional treatment regimens, with this decision being made by pediatric epileptology specialists.

### 2.3. EEG data acquisition and processing

EEG data were collected from 40 ES samples in the study cohort and from 20 age-matched healthy infants. The sample size was chosen after a thorough review of relevant similar studies in previous literature (8, 9). A 19-channel scalp EEG was recorded using the international 10–20 electrode placement system with modified combinatorial nomenclature. An interictal EEG study was conducted for 30 min while subjects were induced into a sleep state using chloral hydrate with a typical oral dose of 50 mg/kg. EEG data were collected during the non-REM (rapid eye movement) sleep stage, as determined by its characteristic features including oculographic findings. A clean, five-minute section devoid of muscle-or eye-related artifacts was manually cropped for analysis. Each subject’s EEG data were carefully reviewed by an epileptology trainee (JK) under the supervision of experienced pediatric neurologists (H-JK, M-JK, and M-SY). The raw EEG data, sampled at a rate of 200 Hz, were re-referenced to a common average reference and preprocessed with a 0.5 Hz low-frequency filter and a 60 Hz notch filter. EEG data for each subject were then segmented into fixed-length epochs of two-second windows without overlap, resulting in 150 epochs being acquired from each of the 40 ES and 20 control subjects. EEG data processing was conducted using MNE and associated packages in Python.

### 2.4. Functional connectivity analysis

To investigate the diverse landscape of spectral power and functional connectivity in individual ES, we conducted a thorough analysis of both frequency and spatial domain characteristics on the prepared EEG data. The frequency range of interest was divided into five bands based on the following boundaries: delta (1–3 Hz), theta (4–7 Hz), alpha (8–13 Hz), beta (14–29 Hz), and gamma (30–80 Hz). For each frequency range, we estimated the individual and cross-spectral density using the multi-taper method. To evaluate functional connectivity, we used the weighted phase-lag index (wPLI), which has been shown to be the most reliable measure in multiple relevant studies (10, 11). The wPLI is a phase-based connectivity measure that assigns greater weight to pairs of series with stronger phase-locking and less weight to smaller phase lags, thereby providing robustness against the influence of common sources, such as volume conduction (12). It is defined by the following equation:


$$wPLI=\frac{\sum Im\left(S_{xy}\right)}{\sum \left|Im\left(S_{xy}\right)\right|}$$

where $Im\left(S_{xy}\right)$ is the imaginary part of the cross-spectral density between the time series x and y .

In this study, we investigated both global and local measures of functional connectivity. Global connectivity was assessed by averaging the wPLI values for all possible connections within the entire set of electrodes. Local connectivity, reflecting short-distance interactions, was evaluated at the level of each individual electrode. This was accomplished by calculating an average of the wPLI values for adjacent (i.e., horizontal, vertical, and diagonal) connections to a given electrode. These connections represent the immediate spatial neighbors of each electrode, providing a measure of local connectivity specific to each anatomical region. Considering hemispheric asymmetries due to potential lateralization of structural abnormality on some subjects, we averaged the measures across both hemispheres (e.g., Fp1/2 represents an average of measurements obtained from electrodes Fp1 and Fp2).

### 2.5. Statistical consideration

Descriptive statistics were used to summarize the baseline characteristics of the cohort and selected sample subjects. All values are presented as medians with interquartile ranges unless otherwise indicated. The distribution of a baseline variable with a standardized mean difference of less than 0.2 between study and control subjects was considered negligible. Differences in EEG metrics between independent subject groups were assessed using the independent t test, while wPLI values were log-transformed to address the skewed distribution (13). A result from the tests was considered statistically significant if the two-tailed *p*-value was less than 0.05. To account for the multiple comparisons inherent in our analysis, the Benjamini-Hochberg correction was applied to the *p*-values. To demonstrate the predictive value of the wPLI connectivity in identifying treatment responders, multivariate logistic regression was utilized for the binary classification of clinical outcomes. The classifiers were selected based on their local connectivity measures, which exhibited significant differences between the treatment response groups. The optimal threshold was determined using the Youden’s J statistic. All computational analyses were performed in Python and R.

## 3. Results

### 3.1. Baseline characteristics

The study cohort comprised 136 ES cases in a single institution between 2002 and 2022 (Table 1). TSC1/2 aberrations were found in 14 (10%) cases, and the majority (63%) showed other structural etiologies. All cases received the standard treatment following electrographic diagnosis, and the interval between age at onset and the initiation of treatment was mostly (80%) within 4 weeks. Vigabatrin was the initial treatment in most cases (80%), whereas some received methylprednisolone pulse therapy followed by oral prednisolone, with or without vigabatrin (14% and 6%, respectively). Most cases showed suppression of spasms and a good response to treatment, but 14 (10%) cases resulted in failure to control spasms and received additional anti-seizure medications (ASMs). Persistent seizures frequently occurred, with 64 (47%) cases requiring two or more ASMs at the last follow-up, and 24 (18%) cases eventually evolved into Lennox–Gastaut syndrome. The cohort was followed until the age of 7.3 (3.4–12.5) years.

**Table 1:** Baseline characteristics and clinical information for the study cohort and sample subjects.

	All ES (*n* = 136)	Study subjects (*n* = 40)
**Age (months)**		
At onset	6.0 (3.0–9.0)	6.5 (4.8–8.3)
At diagnosis^†^	7.5 (5.0–11.3)	8.0 (6.0–11.3)
At treatment^‡^	8.0 (6.0–12.0)	8.0 (6.0–11.3)
**Sex**		
Male	76 (56)	17 (43)
**Etiology**
Non-TSC		
Structural	85 (63)	21 (53)
Genetic	15 (11)	6 (15)
Unknown	22 (16)	10 (25)
TSC	14 (10)	3 (8)
**Initial treatment**		
Vigabatrin	109 (80)	26 (65)
Hormonal therapy	8 (6)	2 (5)
Both	19 (14)	12 (30)
**Clinical outcome**		
Failure to initial treatment	14 (10)	7 (18)
Intractable epilepsy	64 (47)	16 (40)
Lennox–Gastaut syndrome	24 (18)	8 (20)

Values indicate median (IQR) or number (percent). ^†^Electrographic diagnosis. ^‡^Standard treatment (i.e., vigabatrin or hormonal therapy). ES, epileptic spasms; TSC, tuberous sclerosis complex.

For EEG analyses, 40 subjects were selected whose age at study was 8.0 (6.0–11.3) months. Three of the subjects had TSC, while the others had various etiologies, including hypoxic ischemic encephalopathy (*n* = 8), post-hemorrhagic hydrocephalus (*n* = 5), congenital structural anomaly (*n* = 8), trisomy, or other genetic disorders but no remarkable structural abnormalities (*n* = 6), and unknown genetic or structural abnormalities (*n* = 10). Six of the subjects had undergone a neurosurgical shunt procedure for cerebrospinal fluid diversion. None of the subjects were exposed to vigabatrin prior to the EEG study, while 12 had previous neonatal or other focal seizures that were treated with barbiturates (*n* = 9) or other ASMs (*n* = 3). The initial EEG studies presented hypsarrhythmia in all cases, with a BASED score of 4 in 15 cases and 5 in 25 cases. Among the study samples, seven cases were identified as non-responders that failed initial treatment, while the others, including three TSC cases, showed a favorable response to standard treatment and were categorized as responders.

We enrolled 20 age-matched non-epileptic healthy infants without epilepsies or other neurologic conditions as a negative control. The age at EEG recording was 7.0 (4.9–12.1) months with a standardized mean difference of 0.182 from the ES subjects. The control subjects were mostly clinically benign breath-holding spells, and their EEGs were interpreted as normal by pediatric epileptologists.

### 3.2. Distribution of spectral power density

**Figure 2:**
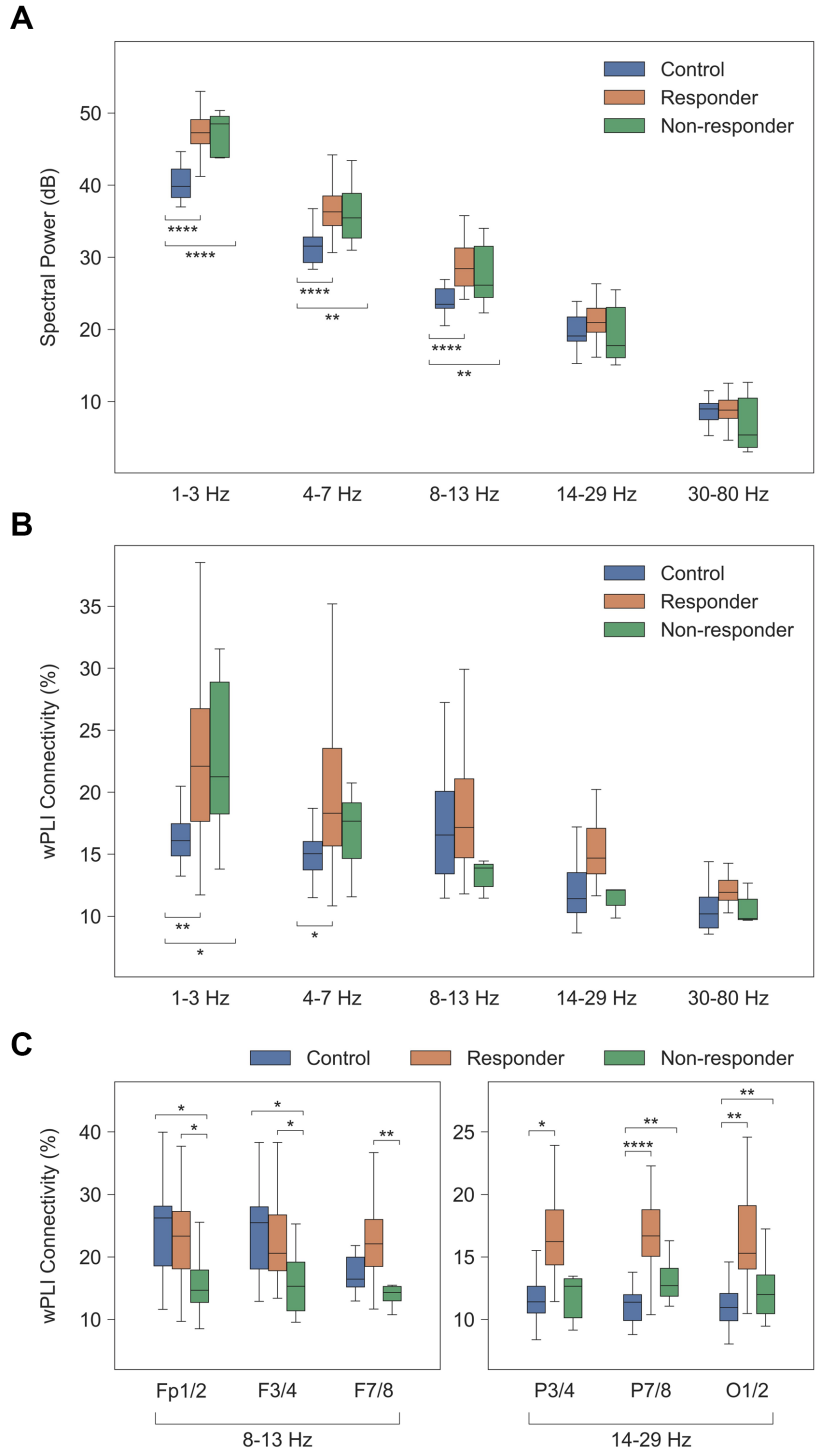
Comparison of computational EEG metrics between healthy controls and treatment groups. Values were averaged across subjects for each group of healthy controls (*n* = 20), treatment responders (*n* = 33), and non-responders (*n* = 7). **(A)** Spectral power density (dB [μV^2^/Hz]). **(B)** Global wPLI connectivity (%). **(C)** Local wPLI connectivity (%) in specific frequency bands. ^*^ *p* < 0.05, ^**^ *p* < 0.01, ^***^ *p* < 1 × 10^−3^, ^****^ *p* < 1 × 10^−4^. wPLI, weighted phase-lag index.

Prior to investigating cross-spectral density and functional connectivity, we assessed the spectral power distribution of each frequency band in the ES and healthy control groups. The spatial distribution of spectral powers thoroughly reflected visual EEG characteristics. ES cases were characterized by diffuse increase in spectral power across all frequency bands, most prominently in the delta and theta bands, possibly due to the typical presentation of hypsarrhythmia in ES. Conversely, the healthy control group exhibited significantly lower spectral power in the delta, theta, and alpha bands compared to the ES group [for delta, theta, and alpha; control vs. responder, *p* = 5.6 × 10^−9^, 2.0 × 10^−6^, and 2.2 × 10^−6^, respectively; control vs. non-responder, *p* = 3.9 × 10^−5^, 1.5 × 10^−3^, and 6.8 × 10^−3^, respectively] (Figure 2A).

### 3.3. Functional connectivity networks

**Figure 3:**
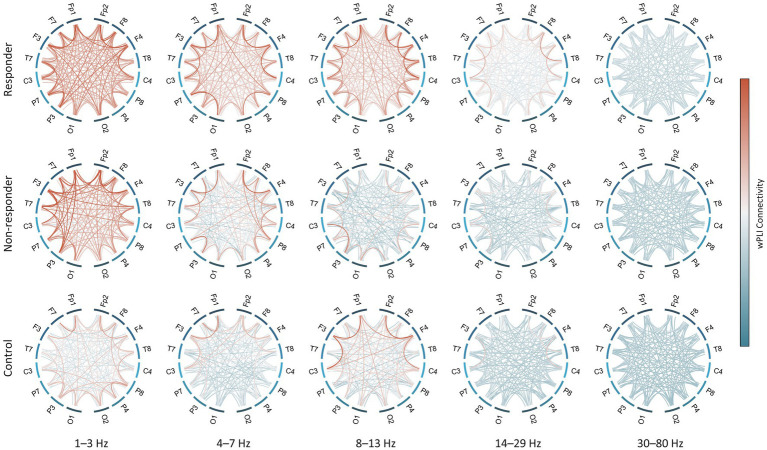
Strength of functional connectivity for each subject group and frequency band. All-to-all connections in both hemispheres are shown except for midline electrodes (i.e., Fz, Cz, and Pz). wPLI values were averaged across subjects in each group. wPLI, weighted phase-lag index.

The global delta wPLI connectivity in healthy controls was significantly lower than in the ES population [control vs. responder, *p* = 2.4 × 10^−3^, and control vs. non-responder, *p* = 0.017] (Figure 2B). This was observed in both intrahemispheric and interhemispheric connections, with ES cases showing notably higher wPLI connectivity in the delta and theta connectivity (Figure 3).

The alpha connectivity showed different spatial patterns in the treatment responder and non-responder groups. The average of short-distance alpha connectivity in non-responders was significantly lower than in responders, particularly in the frontal regions involving the Fp1/2, F3/4, or F7/8 electrodes [responder vs. non-responder, Fp1/2-to-all connections, *p* = 0.026, F3/4, *p* = 0.017, and F7/8, *p* = 0.010] (Figure 2C, left). These characteristics were also significantly different from those of healthy controls except F7/8 [control vs. non-responder, Fp1/2, *p* = 0.016, F3/4, *p* = 0.014, and F7/8, *p* = 0.095].

In addition, treatment responders also showed high beta connectivity in specific regions. The beta connectivity of the posterior electrodes (P7/8 and O1/2) was significantly higher in responders than controls or non-responders [responder vs. non-responder, P7/8, *p* = 0.027 and O1/2, *p* = 0.049; control vs. responder, P7/8, *p* = 2.3 × 10^−7^ and O1/2, *p* = 3.9 × 10^−6^] (Figure 2C, right). However, we found no differences in these connections between non-responders and healthy controls.

### 3.4. Prediction model for clinical responses

**Figure 4:**
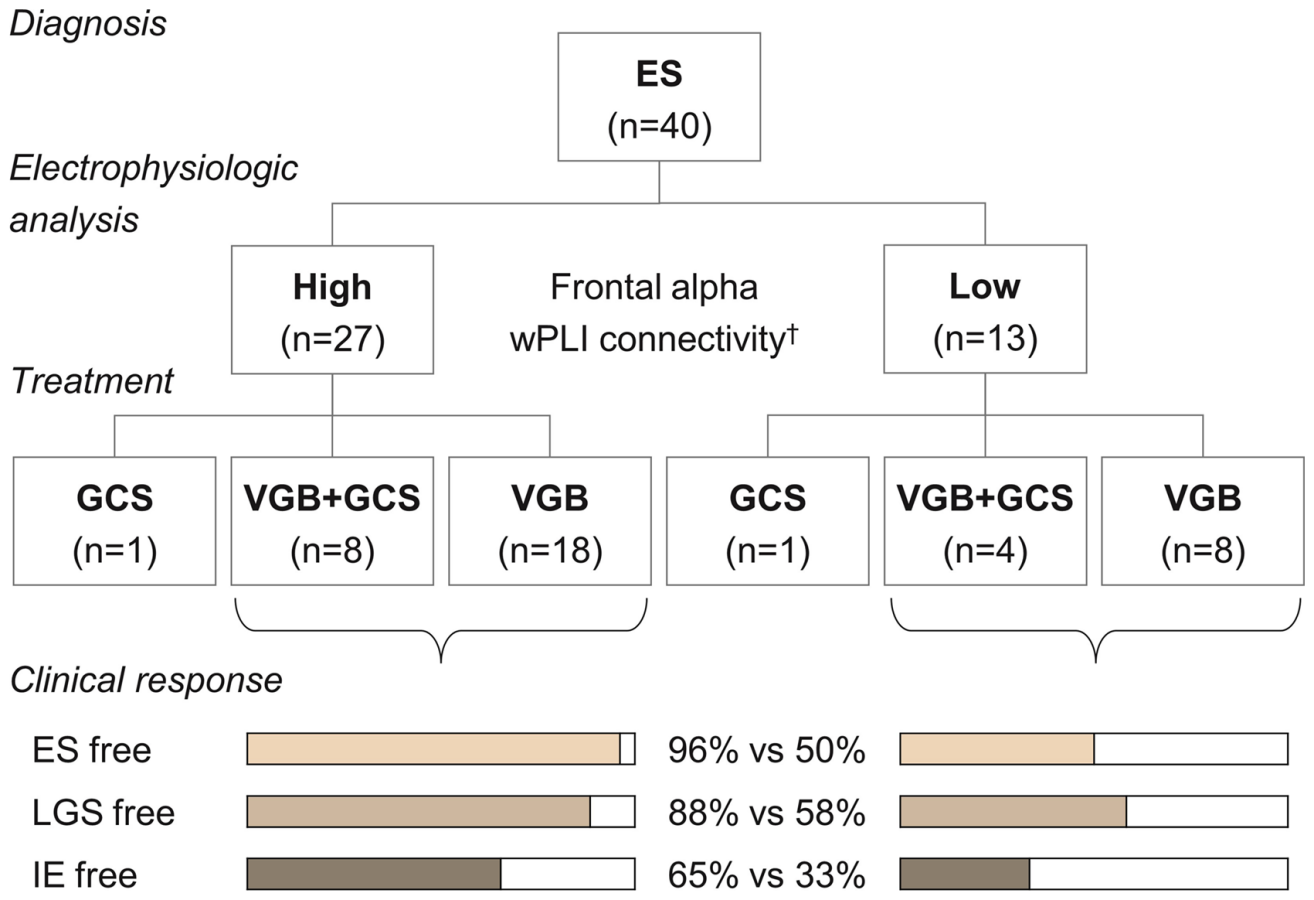
Treatment and clinical responses according to the classification of the demonstrative model. ^†^The alpha connectivity in the frontal regions was estimated using a linear regression model, Y = 1.225 + 0.490 X _Fp1/2_ + 0.456 X _F3/4_ + 0.478 X _F7/8_, and was classified with a threshold of 0.815. ES, epileptic spasms, VGB, vigabatrin, GCS, glucocorticosteroids, LGS, Lennox–Gastaut syndrome, IE, intractable epilepsy.

A demonstrative prediction model using three significant classifiers (i.e., Fp1/2-to-all, F3/4-to-all, and F7/8-to-all alpha connectivity) showed a sensitivity of 0.788 and a specificity of 0.857 for identifying the responder group among ES cases. The overall performance of this model as assessed by the area under the receiver operating characteristic curve was 0.844. When subjects were reorganized according to the model’s prediction scenario (Figure 4), those with higher frontal alpha connectivity showed a good response to vigabatrin with or without hormonal therapy. Conversely, individuals presenting lower alpha connectivity showed unsatisfactory outcomes, with half of the subjects who received vigabatrin treatment failing to control spasms.

### 3.5. Individual patterns of structural and functional brain architectures

**Figure 5:**
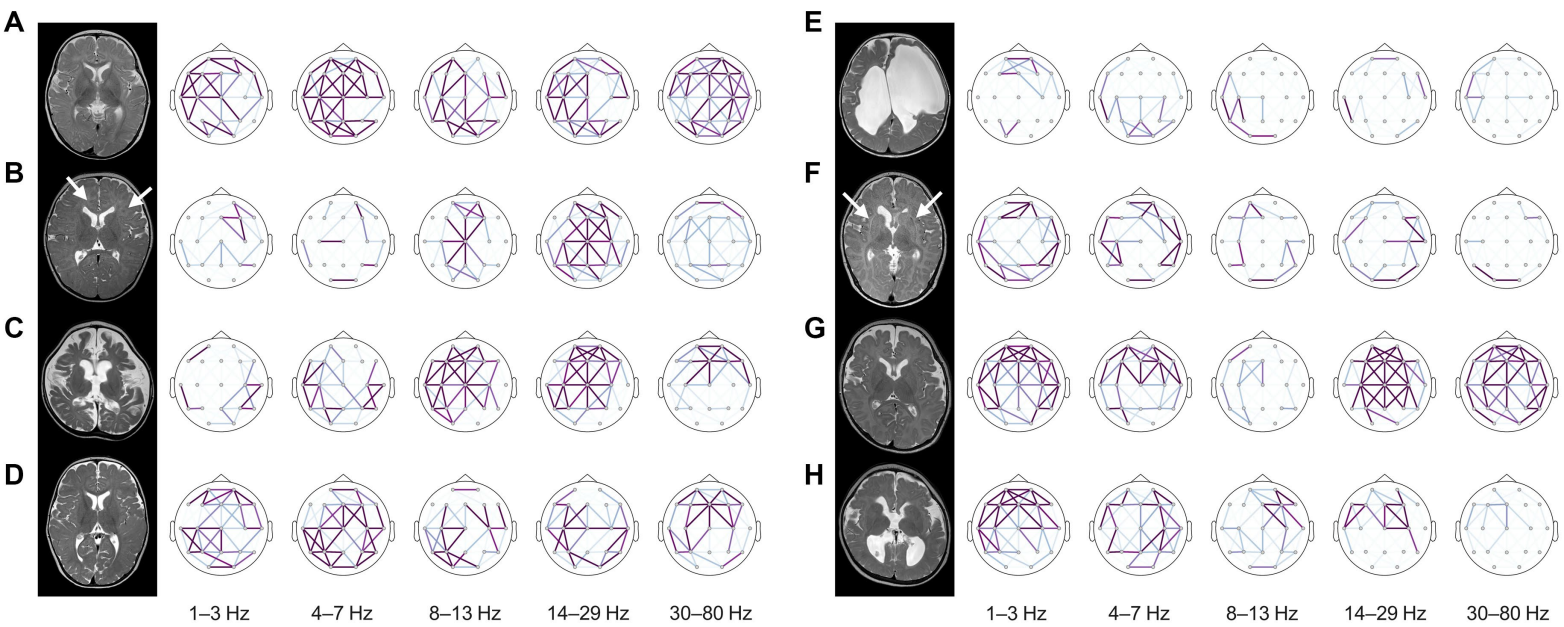
Structural MRIs and brain topography of representative cases. The strength of wPLI connectivity in each frequency band was presented in the topography after standardization using the statistics of healthy controls for each frequency band. The cases on the left side (**A–D**) showed favorable responses to treatment, whereas the cases on the right side (**E–H**) exhibited inadequate control of spasms despite standard treatment. **(A)** A 6-month-old female with unknown etiology showed no structural abnormalities on MRI. Typical characteristics of diffusely increased functional connectivity were presented on the topographic map, and the infant showed a favorable response to vigabatrin monotherapy and was able to develop normally until the last follow-up at eight years old. **(B)** A 6-month-old female with a *TSC2* mutation showed multifocal cerebral lesions (arrows) on MRI. Alpha connectivity was relatively preserved, and the infant showed a good response to vigabatrin monotherapy as expected. **(C)** A 5-month-old female with colpocephaly showed complete agenesis of the corpus callosum and severe diffuse cerebral atrophy. Interestingly, the infant also preserved alpha connectivity in the frontal region and successfully controlled spasms with vigabatrin monotherapy. **(D)** A 7-month-old female with unknown etiology had a good response to vigabatrin monotherapy. This infant preserved global functional connectivity but missed the threshold of the prediction model due to lack of alpha connectivity estimates in the frontal region (false negative). **(E)** A 5-month-old female with an underlying *COL4A1* mutation and porencephaly merely showed functional connectivity. The infant rarely benefited from vigabatrin in combination with methylprednisolone pulse therapy and oral prednisolone. They eventually developed LGS. **(F)** A 3-month-old male with microcephaly showed dysmorphic subcortical and ventricular structures with leukoaraiosis (arrows) resulted from intrauterine insults. The functional connectivity of this subject was globally decreased, and the infant’s spasms were refractory to both vigabatrin and hormonal therapy, evolving into LGS. **(G)** A 6-month-old male with microcephaly showed no remarkable MRI findings on both hemispheres at the onset of spasms. This infant typically lacked alpha connectivity, although connectivity in other frequency bands was preserved. The infant’s spasms were uncontrolled despite combined vigabatrin and hormonal therapy and evolved into LGS accompanied by severe cerebral atrophy on the follow-up MRI. This was diagnosed as alpha-thalassemia X-linked intellectual disability syndrome. **(H)** A 6-month-old female with polymicrogyria and partial agenesis of the corpus callosum had uncontrolled spasms with combined vigabatrin and hormonal therapy. Their alpha connectivity was globally decreased but misclassified by the prediction model due to focal detection of frontal alpha connectivity (false positive). The infant developed LGS, which required a palliative neurosurgical procedure for vagus nerve stimulation. wPLI, weighted phase-lag index, MRI, magnetic resonance image, TSC, tuberous sclerosis complex, LGS, Lennox–Gastaut syndrome.

The patterns of electrophysiologic functional connectivity network in individual subjects were concordant with structural imaging abnormalities (Figure 5). Subjects with normal structures exhibited higher wPLI connectivity, whereas those with destructive cortical and subcortical lesions were associated with lower connectivity within the lesions (Figures 5A,B,E,F). Subjects presenting diffuse encephalomalatic changes in white matter exhibited globally decreased connectivity compared to others. Notably, in some cases, EEG functional connectivity successfully predicted clinical responses that were not demarcated in early imaging studies conducted before complete myelination in infants (Figures 5C,G). It is important to note that some misclassified cases exist due to the limited performance of the prediction model (Figures 5D,H). Overall, these individual functional connectivity patterns implied that subjects with less severe structural abnormalities and higher alpha connectivity are more likely to have a favorable response to treatment.

## 4. Discussion

Several studies have previously investigated functional connectivity networks in ES, suggesting features associated with treatment outcomes (6). It has been consistently reported that ES cases present strong functional connectivity, a conclusion based on results obtained from coherence (14, 15), cross-correlation (8, 9), mutual information (16), and synchronization likelihood (17). Our study replicated these characteristics in ES using a different measure, wPLI, and showed that the strength of functional connectivity, as measured by wPLI, distinctively differentiates ES from age-matched healthy controls.

On the other hand, the existing literature has been inconsistent regarding the use of functional connectivity measures as an EEG marker for the predicting or evaluating clinical responses. Some studies have observed decreased strength of cross-correlation connectivity in ACTH responders (8), whereas others have reported that a greater reduction in coherence connectivity following treatment was associated with a better response to ACTH (7). Another study demonstrated high global wPLI connectivity in delta to alpha frequency bands among ACTH responders (13); however, its scope remained limited due to a lack of validation against healthy controls.

In the current study, we discovered that the strength and spatial distribution of wPLI connectivity were not only different from those found in healthy infants but were also associated with treatment outcomes. Some of our observations in the non-responder group were similar to the decreased beta connectivity patterns found during the ictal stage in previous literature (11), suggesting conceptual consistency. We also noted that lower alpha connectivity in the frontal region was associated with a lower benefit from vigabatrin-based treatment. Additionally, we observed reduced functional connectivity in subjects exhibiting structural abnormalities. This integrative information has provided a better understanding of the structural and functional brain architecture in individual ES cases, which in turn was reflected in their clinical courses.

The prediction model we present in this study showed insufficient sensitivity but readily identified a subset of favorable responders to the current vigabatrin-based treatment. In fact, some subjects did not fit the characteristic alpha connectivity pattern, and our demonstrative model itself was not practical enough for clinical use. Nevertheless, we reveal the potential for this approach to utilize quantitative EEG metrics and a decision tree. As described in previous literature (8), functional connectivity networks derived from computational EEG analysis are not a surrogate for visual EEG features. Despite this, connectivity measures reproduce clinically relevant features that are not easily demarcated by visual EEG assessment. Our results suggest that EEG functional connectivity analysis may be a valuable tool for predicting treatment response and for selecting tailored treatment strategies in individual ES cases. This may therefore lead to improved outcomes for this devastating condition.

Our project was primarily intended to provide an overview of the diverse landscape of computational EEG features. Therefore, the study subjects included individuals with heterogeneous clinical conditions, various etiologies, and treatment modalities. More homogeneous, large cohort studies might yield a higher-performing model, particularly when employing artificial intelligence-based approaches. Our study is also limited for revealing the role of vigabatrin or glucocorticoids in the neural mechanisms implied by functional connectivity networks. Due to limited resources on this project, our study did not include investigation of the longitudinal electrophysiologic changes following treatment. Further studies addressing these aspects are desirable for fully understanding the neurophysiological basis of ES treatment.

## 5. Conclusion

EEG-based functional connectivity networks may provide valuable information for estimating individual treatment responses in ES.

## Data availability statement

The raw data supporting the conclusions of this article will be made available by the authors, without undue reservation.

## Ethics statement

The studies involving human participants were reviewed and approved by the Institutional Review Board of Asan Medical Center, Seoul, Republic of Korea. Written informed consent from the participants' legal guardian/next of kin was not required to participate in this study in accordance with the national legislation and the institutional requirements.

## Author contributions

JK performed data analysis and wrote the original draft of the manuscript. M-JK contributed to data curation. JK, M-JK, and M-SY contributed to conception and design of the study. All authors contributed to the article and approved the submitted version.

## Conflict of interest

The authors declare that the research was conducted in the absence of any commercial or financial relationships that could be construed as a potential conflict of interest.

## Publisher’s note

All claims expressed in this article are solely those of the authors and do not necessarily represent those of their affiliated organizations, or those of the publisher, the editors and the reviewers. Any product that may be evaluated in this article, or claim that may be made by its manufacturer, is not guaranteed or endorsed by the publisher.

## References

[1] Pavone P Polizzi A Marino SD Corsello G Falsaperla R Marino S. West syndrome: a comprehensive review. Neurol Sci. (2020) 41:3547–62. doi: 10.1007/s10072-020-04600-5, PMID: 32827285 PMC7655587

[2] Demarest ST Shellhaas RA Gaillard WD Keator C Nickels KC Hussain SA. The impact of hypsarrhythmia on infantile spasms treatment response: observational cohort study from the National Infantile Spasms Consortium. Epilepsia. (2017) 58:2098–103. doi: 10.1111/epi.13937 29105055 PMC5863227

[3] Hahn J Park G Kang HC Lee JS Kim HD Kim SH. Optimized treatment for infantile spasms: Vigabatrin versus prednisolone versus combination therapy. J Clin Med. (2019) 8:1591. doi: 10.3390/jcm8101591, PMID: 31581698 PMC6832624

[4] Jones K Go C Boyd J Ochi A Mccoy B Puka K. Vigabatrin as first-line treatment for infantile spasms not related to tuberous sclerosis complex. Pediatr Neurol. (2015) 53:141–5. doi: 10.1016/j.pediatrneurol.2015.04.012, PMID: 26227562

[5] Peng P Kessi M Mao L He F Zhang C Chen C. Etiologic classification of 541 infantile spasms cases: a cohort study. Front Pediatr. (2022) 10:774828. doi: 10.3389/fped.2022.774828, PMID: 35330882 PMC8940518

[6] Romero Mila B Remakanthakurup Sindhu K Mytinger JR Shrey DW Lopour BA. EEG biomarkers for the diagnosis and treatment of infantile spasms. Front Neurol. (2022) 13:960454. doi: 10.3389/fneur.2022.960454, PMID: 35968272 PMC9366674

[7] Tanritanir A Vieluf S Jafarpour S Wang X Loddenkemper T. EEG biomarkers of repository Corticotropin injection treatment. J Clin Neurophysiol. (2023) 40:236–43. doi: 10.1097/WNP.0000000000000886, PMID: 34387275

[8] Shrey DW Kim Mcmanus O Rajaraman R Ombao H Hussain SA Lopour BA. Strength and stability of EEG functional connectivity predict treatment response in infants with epileptic spasms. Clin Neurophysiol. (2018) 129:2137–48. doi: 10.1016/j.clinph.2018.07.017, PMID: 30114662 PMC6193760

[9] Smith RJ Hu DK Shrey DW Rajaraman R Hussain SA Lopour BA. Computational characteristics of interictal EEG as objective markers of epileptic spasms. Epilepsy Res. (2021) 176:106704. doi: 10.1016/j.eplepsyres.2021.106704, PMID: 34218209

[10] Haartsen R Van Der Velde B Jones EJH Johnson MH Kemner C. Using multiple short epochs optimises the stability of infant EEG connectivity parameters. Sci Rep. (2020) 10:12703. doi: 10.1038/s41598-020-68981-5, PMID: 32728099 PMC7391718

[11] Zheng R Feng Y Wang T Cao J Wu D Jiang T. Scalp EEG functional connection and brain network in infants with west syndrome. Neural Netw. (2022) 153:76–86. doi: 10.1016/j.neunet.2022.05.029, PMID: 35714423

[12] Vinck M Oostenveld R Van Wingerden M Battaglia F Pennartz CM. An improved index of phase-synchronization for electrophysiological data in the presence of volume-conduction, noise and sample-size bias. NeuroImage. (2011) 55:1548–65. doi: 10.1016/j.neuroimage.2011.01.055, PMID: 21276857

[13] Kanai S Oguri M Okanishi T Miyamoto Y Maeda M Yazaki K. Quantitative pretreatment EEG predicts efficacy of ACTH therapy in infantile epileptic spasms syndrome. Clin Neurophysiol. (2022) 144:83–90. doi: 10.1016/j.clinph.2022.10.004, PMID: 36327598

[14] Burroughs SA Morse RP Mott SH Holmes GL. Brain connectivity in west syndrome. Seizure. (2014) 23:576–9. doi: 10.1016/j.seizure.2014.03.016, PMID: 24794162 PMC4361818

[15] Japaridze N Muthuraman M Moeller F Boor R Anwar AR Deuschl G. Neuronal networks in west syndrome as revealed by source analysis and renormalized partial directed coherence. Brain Topogr. (2013) 26:157–70. doi: 10.1007/s10548-012-0245-y, PMID: 23011408

[16] Davis PE Kapur K Filip-Dhima R Trowbridge SK Little E Wilson A. Increased electroencephalography connectivity precedes epileptic spasm onset in infants with tuberous sclerosis complex. Epilepsia. (2019) 60:1721–32. doi: 10.1111/epi.16284 31297797 PMC6687536

[17] Suzuki H Otsubo H Yokota N Nishijima S Go C Carter Snead O. Epileptogenic modulation index and synchronization in hypsarrhythmia of west syndrome secondary to perinatal arterial ischemic stroke. Clin Neurophysiol. (2021) 132:1185–93. doi: 10.1016/j.clinph.2020.12.028, PMID: 33674213

